# Effects of Malondialdehyde on Growth Performance, Gastrointestinal Health, and Muscle Quality of Striped Catfish (*Pangasianodon hypophthalmus*)

**DOI:** 10.3390/antiox13121524

**Published:** 2024-12-13

**Authors:** Cong Peng, Xinlangji Fu, Yumeng Zhang, Haitao Zhang, Yuantu Ye, Junming Deng, Beiping Tan

**Affiliations:** 1 College of Fisheries, Guangdong Ocean University, Zhanjiang 524088, China; congpeng@stu.gdou.edu.cn (C.P.); 2112001038@stu.gdou.edu.cn (X.F.); 2112001111@stu.gdou.edu.cn (Y.Z.); 2 Key Laboratory of Aquatic, Livestock and Poultry Feed Science and Technology in South China, Ministry of Agriculture, Zhanjiang 524088, China; 13828263599@139.com; 3 Guangdong Evergreen Feed Industry Co., Ltd., Zhanjiang 524022, China; 4 School of Biology & Basic Medical Science, Soochow University, Suzhou 215123, China; yeyt@suda.edu.cn

**Keywords:** malondialdehyde, gastrointestinal injury, intestinal inflammation, intestinal barrier, muscle quality

## Abstract

Malondialdehyde (MDA) is a reactive carbonyl compound produced through lipid peroxidation during feed storage, which poses a significant threat to fish health. This study aimed to evaluate the effects of dietary MDA on the growth rate, gastrointestinal health, and muscle quality of striped catfish (*Pangasianodon hypophthalmus*). A basal diet (M0) containing 34% crude protein and 10.5% crude lipid was formulated. Each group was sprayed with malondialdehyde solution (0, 5, 10, 20, 40, and 80 mg/kg, on dietary crude lipid basis; 0, 0.53, 1.07, 2.13, 4.26, and 8.52 mg/kg, on dietary basis) before feeding, respectively. Each diet was randomly assigned to triplicates of 30 striped catfish (initial weight 31.38 g) per net cage. After 8 weeks, dietary inclusion of MDA regardless of level significantly depressed the growth rate and feed utilization. The extent of structural damage to the gastrointestinal tract increased progressively with increasing dietary MDA levels. The extent of damage to the intestinal biological barrier (intestinal microbial structure), chemical barrier (trypsin, lipase, amylase, and maltase activity), physical barrier (*zonula occludent-2*, *occludin*, *claudin 7α*, and *claudin 12* relative expression), and immune barrier (contents of complement 4, complement 3, immunoglobulin M, and lysozyme activity) was dose-related to dietary MDA. Moreover, a linear decline in the activities of intestinal antioxidant enzymes (catalas, superoxide dismutase, et al.) and anti-inflammatory factor (*transforming growth factor beta1*, *interleukin 10*) relative expression was noted alongside an increase in dietary MDA content. In contrast, the relative expression levels of intestinal inflammatory factor (*interleukin 8*, *transcription factor p65*, *tumor necrosis factor alpha*) relative expression displayed an opposing trend. Additionally, dietary MDA exerted a linear influence on muscle color and texture characteristics. In conclusion, high doses of MDA (5–80 mg/kg) reduced the growth performance of striped catfish, attributed to linear damage to the gastrointestinal tract, a linear decrease in antioxidant function, and the occurrence of an inflammatory response. High doses of MDA (>40 mg/kg) were observed to significantly increase dorsal muscle b-value and induce muscle yellowing.

## 1. Introduction

Green aquaculture plays a vital role in providing healthy and high-quality proteins to meet the needs of a growing global population in an environmentally sustainable manner [1,2,3]. The quality of aquatic products, a concern shared worldwide, is intricately tied to the quality of the feed they receive. Therefore, it is essential for the continued health and sustainability of aquaculture practices to ensure the safety of feed [4,5]. Lipids, crucial for supplying fatty acids and energy to fish, are key macronutrients in their diet [6]. Numerous studies have underscored the detrimental effects of dietary lipid oxidation, particularly from oxidized fish oil, on various aspects of aquatic organisms such as growth performance [7], antioxidant function [8,9], liver health [10,11], and muscle quality [12,13]. Malondialdehyde (MDA), a byproduct of lipid oxidation, is known for its toxic effects on proteins [14,15] and DNA [16] due to its molecular structure. It is present in almost all fat-containing feeds and is often used to assess the degree of oxidative deterioration of feeds. What is more, the main risks associated with MDA in aquaculture include the disruption of essential nutrient structures in feed, such as rice bran [17], sunflower oil [18], and soybean meal [19], which are gradually being reported. Consequently, the potential harm of MDA produced by oxidized lipids to aquaculture should not be ignored, and aquaculture requires a blueprint to perfect the toxic mechanisms of MDA.

The striped catfish (*Pangasianodon hypophthalmus*), also known as the “tra catfish”, is a tropical freshwater catfish commonly found in the bottom water with unique economic value in the Mekong River Basin [20,21]. It is processed into frozen fillets and fish oil for its high-quality white meat and high lipid content. It has a huge market and is often exported to 145 countries [22,23]. In addition, the striped catfish is extensively cultured for its rapid growth, adaptability, and tolerance to low oxygen levels [24]. In 2018, the striped catfish production reached 2.36 million tons (4.3% of world freshwater fish production), making it the tenth largest finfish producer fish in the world [25]. With the development of the aquaculture industry, its annual production in 2020 reached 2.52 million tons, valued at around $3 billion [26,27]. China has the largest import market for striped catfish in the world, and it remains a major aquaculture country with a huge average annual production (36% of total aquatic animal production) [28]. The striped catfish farming industry initially developed in regions like Guangdong, Hainan, and Guangxi in South China, establishing a comprehensive industrial chain from breeding to sales [29]. However, the rapid expansion of striped catfish farming in China has found issues such as digestive tract damage and muscle yellowing, impacting product quality and causing significant economic losses [30,31,32]. The monsoon climate of Bangladesh exhibits similarities to that of China, and the phenomenon of yellow muscle was also a common occurrence [33]. This climate, characterized by high temperatures and humidity, is well known to readily induce lipid peroxidation, leading to the formation of reactive carbonyl compounds, including MDA [34]. Muscle yellowing is intimately associated with lipid oxidation and oxidative stress [35]. MDA has been shown to cause the accumulation of intracellular reactive oxygen species, mitochondrial dysfunction, and oxidative stress [36]. It can be hypothesized that MDA may be a potential factor contributing to digestive tract damage and muscle yellowing in striped catfish. According to the national standard, the MDA content limit in the compound feed of striped catfish is 5.0 mg/kg (on a dietary crude lipid basis) [37]. In addition, a higher content of MDA (8.65 mg/kg, on crackers basis) was determined in crackers containing oat and linseed oil and stored [38]. Consequently, the striped catfish may face dietary challenges with high doses of MDA. The aim of this study was to investigate the effects of high levels of MDA in diets on the growth, gastrointestinal health, and muscle quality of striped catfish. This can complete the blueprint of the MDA toxicity mechanism in aquaculture and offer a theoretical foundation for the safety limit of MDA content in the diet for striped catfish.

## 2. Materials and Methods

### 2.1. Preparation of MDA Solution

In accordance with the National Standard [39], the MDA standard stock solution (100 μg/mL) was prepared by dissolving 0.315 g of 1,1,3,3-tetraethoxypropane (purity ≥ 97%, Maclin Biochemical Technology Co., Ltd., Shanghai, China) in double-distilled water and then diluting the solution to a final volume of 1000 mL. The MDA standard stock solution was maintained at 4 °C, and the corresponding concentration of the MDA use solution was formulated based on the lipid content of the feed.

### 2.2. Experimental Diets

A diet (34% crude protein, 10.5% crude lipid) was formulated with white fish meal as the main protein source and soybean oil as the lipid source (Table 1). Six groups were designed for the experiment. The MDA solution for each group was calculated and prepared based on the crude lipid content of the diet required for each day. The MDA solutions prepared were equal volumes of different concentrations (0, 5, 10, 20, 40, and 80 mg/kg, based on crude dietary lipid; 0, 0.53, 1.07, 2.13, 4.26, and 8.52 mg/kg, on a dietary basis). The MDA solution was sprayed evenly on the surface of the feed, mixed, dried, and immediately fed to the striped catfish.

Except for the lipid sources, all of the ingredients were ground and mixed in accordance with the methodology outlined by Zhang et al. [40]. Feed preparation and storage procedures were determined according to Liu et al. [41]. Proximate composition analysis on feed ingredients and experimental diets were performed following standard laboratory procedures [42].

### 2.3. Feeding Management

The experiment was conducted at the Freshwater Base laboratory (Guangdong Ocean University, Zhanjiang, China). Approximately 18 rectangular net cages (0.7 × 0.7 × 1 m^3^) were set up in a rectangular tank (5 × 5 × 2 m^3^) on the Freshwater Base laboratory. Each group was randomly assigned three net cages, with each net cage containing 30 fish. Each net cage was spaced more than 20 cm apart to ensure adequate separation. Experimental juvenile striped catfish were sourced from a local commercial fish farm (Hengxing Feed Co., Ltd., Zhanjiang, China). All fish were placed in a rectangular tank and fed a test diet for a period of two weeks to acclimatize to the experimental conditions, followed by a 24 h fasting period. Subsequently, fish with an initial weight of 31.38 ± 0.09 g were randomly selected and divided into 18 rectangular net cages. The feeding process was completed manually and slowly, with the feed provided in three rounds until the fish were satiated. Upon completion of the feeding procedure, no feed deposits were observed within the net cages. The fish were fed twice a day (at 07:00 and 17:00) for eight weeks. Additionally, all net cages received continuous aeration and were maintained under natural photoperiod conditions. During the experimental period, the water temperature was maintained at 25–30 °C, pH 7.7–8.0, ammonia nitrogen < 0.2 mg/L, and dissolved oxygen > 6.0 mg/L.

### 2.4. Sample Collection

Prior to sampling, fish in each net cage were deprived of feed for 24 h and anesthetized using an eugenol solution (1:12,000; Shanghai Maclin Biochemical Technology Co., Ltd., Shanghai, China). After that, fish were weighed to assess the growth performance and feed utilization. Three fish were randomly selected from each net cage and dissected to measure individual body weight and length; visceral mass, liver, intestinal weight, and intestinal length were separated and measured separately to analyze morphological parameters. Additionally, six fish per net cage were chosen for blood sample collection following the method described by Deng et al. [43]. These fish were subsequently dissected, and their stomachs, foregut, and hindgut were removed for enzyme activity analyses. The rest dorsal muscles of the fish were cut to a standardized size (2 × 1 × 1 cm^3^) for the analysis of muscle color and textural characteristics. Four fish were randomly caught from each net cage, dissected, and their hindgut placed in Eppendorf (EP) tubes containing RNA later for RNA extraction. Three stomachs and hindguts per net cage were placed in EP tubes containing 4% paraformaldehyde for hematoxylin-eosin (H&E) staining. Moreover, the stomach and hindgut of two fish from the M0, M20, and M80 groups were placed in EP tubes with a 2.5% glutaraldehyde solution for scanning electron microscope (SEM) sections of stomach and transmission electron microscope (TEM) sections of intestine. Twelve hindguts of fish from each group were placed in four EP tubes, quickly frozen in liquid nitrogen, and then transferred to −80 °C for storage. Four EP tubes were mixed into three mix samples and were utilized for intestinal microbial analysis. All the dissections were performed on a low-temperature sterile bench to avoid sample contamination. All the samples were mixed prior to further analysis.

### 2.5. Gastrointestinal Tract Histological Examination

H&E-stained sections of the stomach were prepared following the methodology outlined by Li et al. [44]. Quantitative histomorphometric measurements of stomach and intestinal villi height, villi width, and muscular layer thickness were performed using NIH ImageJ software (software version 2.3.0/1.53f). Similarly, intestinal H&E-stained sections were prepared in accordance with the procedures established by Li et al. [45]. Additionally, stomach SEM and intestinal TEM sections were prepared as described by Huang et al. [46].

### 2.6. Intestinal Digestive Enzyme Activity

The samples of stomach and foregut were mixed separately with nine times the volume of ice saline and homogenized using a homogenizer (IKA-Werke GmbH and CO. KG, Staufen, Germany). Subsequently, the supernatant was collected after centrifugation (4 °C, 10 min, 3500× *g*). The activities of pepsin and intestinal trypsin, lipase, amylase, maltase, and total protein (TP) content were measured using commercial kits (Nanjing Jiancheng Bioengineering Institute, Nanjing, China). The determination process of each index was carried out in strict accordance with the corresponding instructions, and the corresponding units were converted (the same as below).

### 2.7. Biochemical Indicators in Serum and Intestinal Immune Factor Contents

Catalase (CAT), superoxide dismutase (SOD), glutathione peroxidase (GPx), glutathione reductase (GR), and peroxidase (POD) activities, total antioxidant capacity (T-AOC), TP, and MDA content were measured using reagent test kits (Nanjing Jiancheng Bioengineering Institute, Nanjing, China).

The serum and intestinal contents of complement 3 (C3), complement 4 (C4), immunoglobulin M (IgM), and lysozyme (LYZ) activity were measured using commercial kits (Shanghai Enzyme Linked Biotechnology Technology Co., Ltd., Shanghai, China). The serum content of lipopolysaccharide (LPS) and diamine oxidase (DAO) activity was also measured using commercial kits.

### 2.8. Intestinal Microbial Composition

Total genomic DNA was extracted using the HiPure Stool DNA kit (Magen Biotech-nology Co., Ltd., Guangzhou, China). Afterwards, the V3–V4 region of the bacterial 16S rRNA gene fragment was amplified using a universal primer for the 16S rRNA gene fragment (341F: CCTACGGGGNGGCWGCAG; 806 R: GGACTACHVGGGGTATCTAAT). In addition, amplicons were collected from 2% agarose gels, purified using the AxyPrep DNA Gel Extraction Kit (Axygen BioScience Inc., Union City, CA, USA), and quantified using the ABI StepOnePlus Real-Time PCR System (Life Technologies Inc., Foster City, CA, USA). Finally, purified amplicons were paired-end sequenced on the Illumina platform (Illumina Inc., San Diego, CA, USA). The raw data were analyzed on the Omicsmart platform (http://www.omicsmart.com).

### 2.9. Quantitative PCR Measurement

Total intestinal RNA was extracted by a commercially available TransZol Up Plus RNA Kit (All-style Gold Biotechnology Co., Ltd., Beijing, China), followed by concentration and quality control using a spectrophotometer. Subsequently, cDNA was synthesized from qualified RNA samples using the Prime Script™ RT reagent Kit (Takara Bio Inc., Beijing, China). Gene-specific primers were designed using Primer 5 software based on the sequences in Gene Bank (Table 2). The 10 μL RT-qPCR reaction volume contained 5 μL SYBR^®^ Premix ExTaq™ II, 3.2 μL sterile water, 1 μL cDNA, and 0.4 μL each of the upstream and downstream primers. The RT-qPCR reaction conditions were as follows: 30 s at 95 °C (pre-denaturation), 5 s at 95 °C (denaturation), and 34 s at 60 °C (extension) for 40 cycles. The 2^−ΔΔCT^ method was used to calculate relative gene expression results [47].

### 2.10. Muscle Color and Texture Characteristics Analysis

The color of the dorsal muscles was measured with the Minolta colorimeter CR400 (Konica Minolta Holdings, Inc., Tokyo, Japan). Muscle color was quantified according to the CIE color specification system. The system includes L* describing lightness (L* = 0 for black, L* = 100 for white), a* describing intensity in red (a* > 0), and b* describing intensity in yellow (b* > 0). All measurements were conducted under standard D65 light source conditions. Furthermore, the texture characteristics were analyzed using the TA.XT plus C texture analyzer (Stable Micro system Ltd., Surrey, UK).

### 2.11. Calculations and Statistical Analysis

Normality and variance homogeneity assumptions were confirmed prior to any statistical analysis. All evaluated variables were analyzed using a one-way ANOVA with Tukey’s HSD test to determine whether the inclusion levels of MDA significantly affected the observed responses (*p* < 0.05). In addition, the simple linear regression model contrasts across graded levels of MDA were used to evaluate the responses for all dependent variables, the statistical methodological reference from Hansen et al. [48]. The determination (R^2^) was calculated. All statistical analysis was performed using SPSS 22.0 for Windows (SPSS Inc., Chicago, IL, USA).

## 3. Results

### 3.1. Growth Performance

The growth rate and feed utilization were reduced by dietary MDA (Table 3). Fish in the M0 group exhibited higher final body weight (FBW), weight gain ratio (WGR), protein efficiency ratio (PER), and intestinal weight index (ISI) than others (*p* < 0.05). Compared to the M5, M10, and M20 groups, specific growth rate (SGR) was significantly higher in the M0 group (*p* < 0.05). There was a linear increase in the hepatosomatic index (HSI) (*p* < 0.05). Interestingly, the intestinal length index (ILI) and ISI showed the opposite trend (*p* < 0.05). The dietary MDA level had no significant effect on viscerosomatic index (VSI) or condition factor (CF) in the experimental groups.

### 3.2. Digestion and Absorption Function

**Figure 1:**
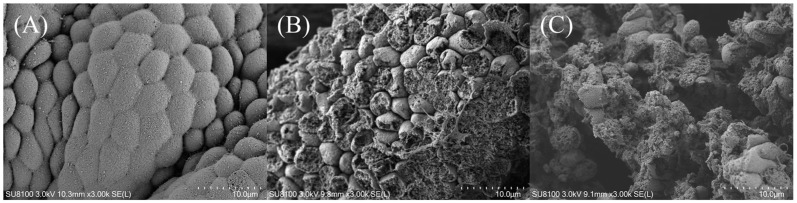
The scanning electron microscope of the stomach in striped catfish-fed diets with various levels of malondialdehyde (×3000). (**A**) Diet M0, (**B**) Diet M20, (**C**) Diet M80. Striped catfish-fed the M0 diet (**A**) showed the stomach mucosal surfaces were smooth, soft, and free of erosions, while stomach mucosal cells were ruptured, and the mucosa was extensively ulcerated in fish fed the M20 (**B**) and M80 (**C**) diets.

The stomach mucosa of the M0 group displayed a smooth surface, intact cellular structure, clear boundaries, and a tight arrangement of cells. As the dietary MDA content increased, the stomach mucosa exhibited a gradual loss of integrity, manifesting as ulcerated surfaces of varying sizes and irregular shapes, as well as a disruption of the cell structure (Figure 1).

**Figure 2:**
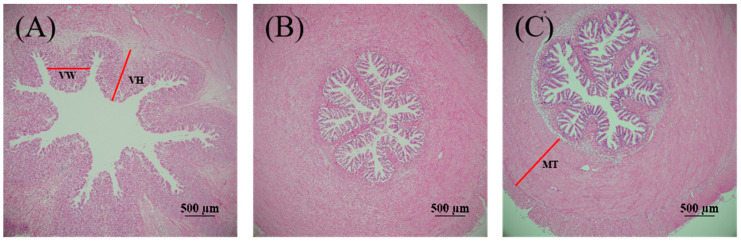

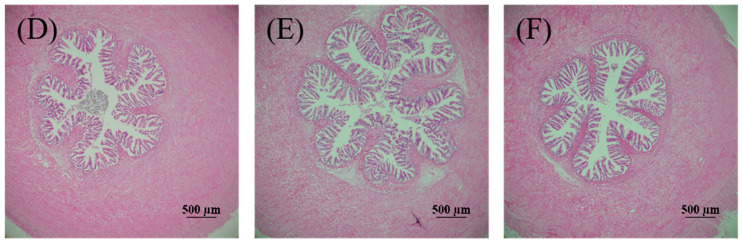
The stomach histomorphology of striped catfish-fed diets with various levels of malondialdehyde (H&E staining, ×40). (**A**) Diet M0, (**B**) Diet M5, (**C**) Diet M10, (**D**) Diet M20, (**E**) Diet M40, (**F**) Diet M80. VH, villi height; VW, villi width; MT, muscular layer thickness. Striped catfish-fed the M0 (**A**) and M5 (**B**) diets exhibited healthy stomach structure with intact columnar epithelium, stomach glands, mucosa, and submucosa, while damaged stomach tissue with the degenerated columnar epithelium, atrophied stomach glands, and destructed villus integrity was observed in striped catfish fed the M10 (**C**), M20 (**D**), M40 (**E**), and M80 (**F**) diets.

**Figure 3:**
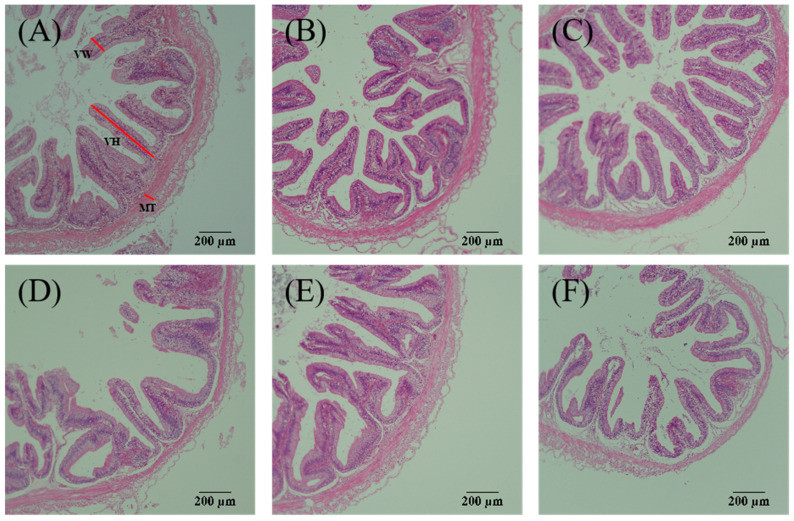
The intestinal histomorphology of striped catfish-fed diets with various levels of malondialdehyde (H&E staining, ×100). (**A**) Diet M0, (**B**) Diet M5, (**C**) Diet M10, (**D**) Diet M20, (**E**) Diet M40, (**F**) Diet M80. VH, villi height; VW, villi width; MT, muscular layer thickness. Striped catfish-fed the M0 (**A**) and M5 (**B**) diets exhibited normal intestines with intact villus, while the damaged intestine with shortened villus and thinned lamina propria was observed in fish fed the M10 (**C**), M20 (**D**), M40 (**E**), and M80 (**F**) diets.

Furthermore, the stomach H&E staining allows several key features to be observed, including villus width, villus height, and muscular layer thickness (Figure 2). Based on statistical analysis, a linear response was found for stomach villus width, villus height, and muscular layer thickness, which attenuated with increasing MDA levels in the diets (*p* < 0.05; Table 4). Observations from H&E staining confirmed that dietary intake of 10–80 mg/kg MDA damaged villus width, villus height, and muscular layer thickness in the intestine (Figure 3). Based on statistical analysis, the relationship between dietary MDA intake and the dependent variables intestine villus height, villus width, and muscular layer thickness was best explained by a linear model (*p* < 0.05; Table 4).

There was a linear decline in the activities of digestive enzymes, including trypsin, lipase, amylase, and maltase (*p* < 0.05). However, no significant effect was observed for pepsin (Table 5).

### 3.3. Intestinal Mucosal Barrier

#### 3.3.1. Intestinal Mucosal Permeability

A linear increase in serum DAO activity and LPS levels was observed with increasing dietary MDA content (*p* < 0.05; Table 6).

#### 3.3.2. Intestinal Biological Barrier

**Figure 4:**
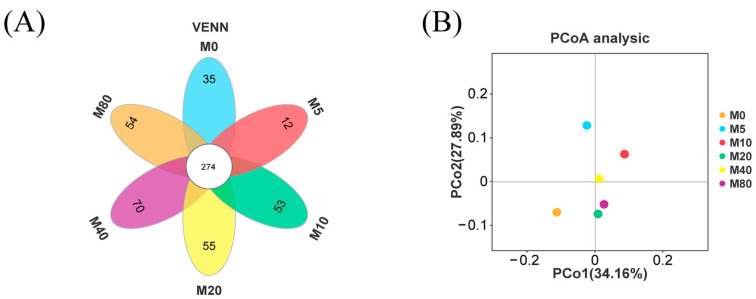
The intestinal microbial diversity of striped catfish-fed diets containing different levels of malondialdehyde. (**A**) Venn diagram based on the OTU level (each group is represented by a different color. The intersection part of the figure represents the common OTUs between different groups), (**B**) Principal Co-ordinates analysis diagram based on the OTU level and weighted_unifrac Distance (The dots represent a group, PCoA1 represents the principal coordinate component that best explains the variation in the data, and PCoA2 represents the principal coordinate component that accounts for most of the remaining variation).

A total of 1631 Operational taxonomic units (OTUs) were generated after noise reduction and data flattening. Approximately 274 common OTUs were annotated between all groups, whereas 35, 12, 53, 55, 70, and 54 unique OTUs existed in the M0, M5, M10, M20, M40, and M80 groups, respectively (Figure 4A). After calculating the alpha diversity of the groups (Table 7), it was found that the level of dietary MDA inclusion had no relationship with the estimators of community richness (Chao and Ace) and diversity (Shannon and Simpson). In addition, Principal Co-ordinates analysis showed that the microbial structures of the M10, M20, M40, and M80 groups are similar, while the M0 and M5 groups were relatively distant (Figure 4B).

**Figure 5:**
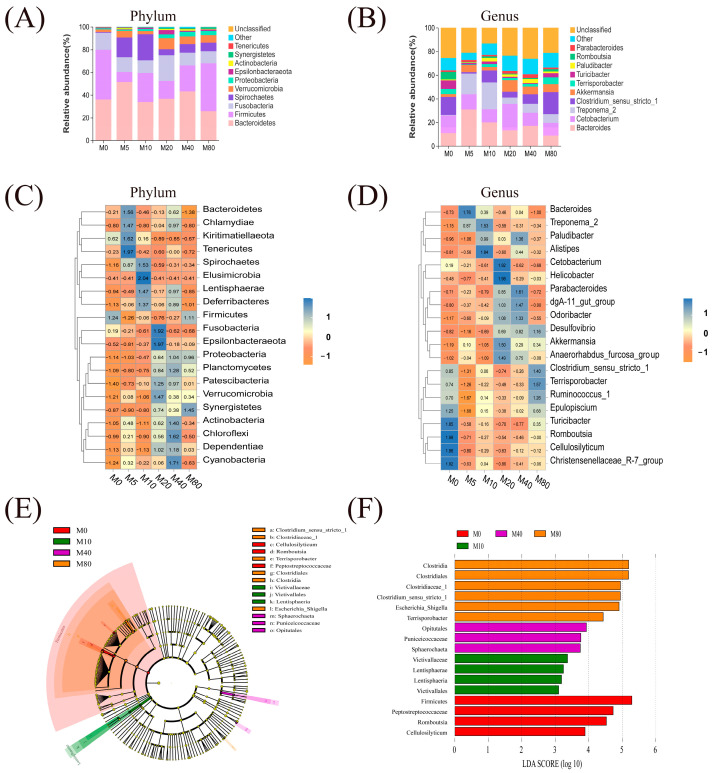
The intestinal microbial composition of striped catfish-fed diets containing different malondialdehyde levels. (**A**) Plot of microbial composition based on the phylum level; (**B**) Plot of microbial composition based on the genus level; (**C**) Heat map of microbial abundance based on the phylum level; (**D**) Heat map of microbial abundance based on the genus level. (**C**,**D**) each column represents a group; each row represents a species. The colors represent species abundance; nearer to orange is less abundance and nearer to blue is more abundance. (**E**) Evolutionary clade chart, (**F**) LDA distribution histogram (**E**) Illustrating the differential species across various taxonomic ranks, from inner to outer in the following sequence: Phylum, Class, Order, Family, Genus, Species. (**F**) bacterial taxa differentially represented in the intestinal microbial populations of different groups were identified by LEfSe using an LDA, with LDA scores > 3 and *p* < 0.05).

Bacteroidetes, Firmicutes, Fusobacteria, Spirochaetes, Verrucomicrobia, Proteobacteria, Epsilonbacteraeota, Actinobacteria, Synergistetes, and Tenericutes were the most abundant phylum levels of richness in all groups (Figure 5A). *Bacteroides*, *Cetobacterium*, *greponema_2*, *Clostridium_sensu_stricto_1*, *Akkermansia*, *Terrisporobacter*, *Turicibacter*, *Paludibacter*, *Romboutsia*, and *Parabacteroides* were the most abundant genus-level of richness in all groups (Figure 5B). Furthermore, heat maps were created at the phylum and genus level. These were used to identify similarities and differences in community composition among different samples (Figure 5C,D). Moreover, linear discriminant analysis (LDA) was performed to screen for differences in dominant microbes among all groups. It was shown that M5 and M20 had no advantage over the other four groups. The M0, M10, M40, and M80 groups exhibited 4 (Firmicutes, Peptostreptococcaceae, *Cellulosilyticum*, *Romboutsia*), 4 (Lentisphaerae, Lentisphaeria, Victivallales, Victivallaceae), 3 (Opitutales, Sphaerochaeta, *Puniceicoccaceae*), and 6 (Clostridia, Clostridiales, Clostridiaceae_1, *Terrisporobacter*, *Escherichia_Shigella*, *Clostridiun_sensu_stricto_1*) biomarkers with significantly higher relative abundance, respectively (Figure 5E,F).

**Figure 6:**
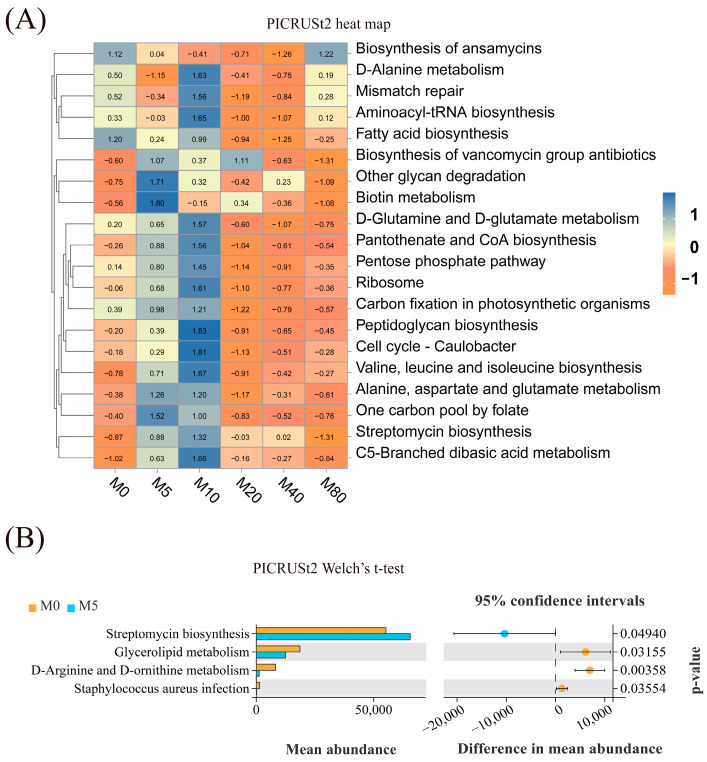
The intestinal microbial functional prediction at the species level in striped catfish-fed diets with various malondialdehyde levels. (**A**) PICRUSt2 heat map of third-level functional pathway annotation (each column represents a group; each row represents a functional pathway. The colors represent the relative abundance of the pathway; the warmer the color (closer to orange), the less the abundance; the cooler the color (closer to blue), the more the abundance). (**B**) PICRUSt2 Welch’s t-test of third-level functional pathway annotation (significant when *p* < 0.05).

Functional prediction on the Kyoto Encyclopedia of Genes and Genomes database was annotated based on 16S sequencing data. The heat maps were used to show similarities and differences in functional pathway annotation among different samples (Figure 6A). Furthermore, the abundance of D-arginine and D-ornithine metabolism, glycerolipid metabolism, and staphylococcus aureus infection significantly decreased in the M5 group compared with the M0 group (*p* < 0.05). Conversely, the abundance of streptomycin biosynthesis decreased significantly (*p* < 0.05; Figure 6B).

#### 3.3.3. Intestinal Physical Barrier

**Figure 7:**
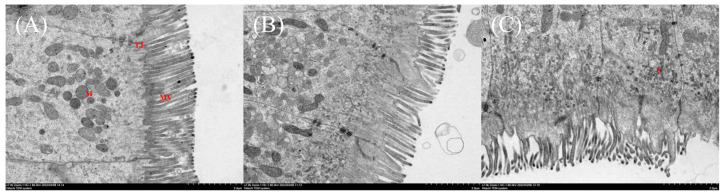
The scanning electron microscope of the intestine in striped catfish-fed diets with various levels of malondialdehyde (×7000). (**A**) Diet M0; (**B**) Diet M20; (**C**) Diet M80. MV, microvilli; M, mitochondria; TJ, tight junction; L, lysosome. Striped catfish-fed the M0 diet (**A**) showed normal enterocytes, while enterocytes with sparse and disorganized microvilli, swollen mitochondria, and widened intercellular space were observed in fish fed the M20 (**B**) and M80 (**C**) diets.

**Figure 8:**
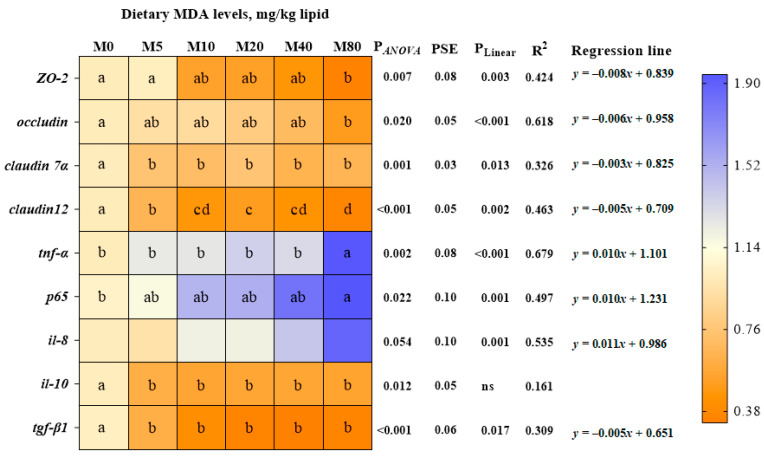
The relative expression of inflammatory response and tight junction protein-related genes in striped catfish-fed diets with various levels of malondialdehyde (*n* = 6). Values are presented as means of triplication; means in the same row with different superscript letters represented a significant difference (*p* < 0.05); PSE = pooled standard error of means. *ZO-2*, zonula occludent 2; *tnf-α*, tumor necrosis factor alpha; *p65*, transcription factor p65; *il-8*, interleukin 8; *il-10*, interleukin 10; *tgf-β1*, transforming growth factor beta1. Linear regression equation (where y is the response and x is the level of malondialdehyde in diet), R^2^, and *p*-Value (significant when *p* < 0.05) are also given.

The intestinal mucosal cells of striped catfish in the M0 group were observed to be closely connected and displayed a complete cell structure. However, there was a gradual disorganization of the intestinal microvilli, damage to the cell membrane structure, and dissolution of the organelles with increased dietary MDA content (Figure 7). In addition, the mRNA relative levels of zonula occludent 2 (*ZO-2*), *occludin*, *claudin 7α*, and *claudin 12* exhibited a linear decline in concentration with elevated dietary MDA levels (*p* < 0.05; Figure 8).

#### 3.3.4. Intestinal Immune Barrier

A linear decline was observed for intestinal contents of C3, C4, and IgM, as well as the activities of LYZ, POD, CAT, SOD, and GR (*p* < 0.05; Table 8 and Table 9). Contrarily, the intestinal MDA content showed the opposite trend (*p* < 0.05). No significant effect was observed for the serum C3, C4, LYZ, and IgM contents in fish fed the experimental diets. The intestinal T-AOC also showed no significant difference. Additionally, there was a linear decrease in intestinal transforming growth factor beta 1 (*tgf-β1*) relative expression with increasing dietary MDA (*p* < 0.05). Conversely, intestinal interleukin 8 (*il-8*), tumor necrosis factor-alpha (*tnf-α*), and transcription factor p65 (*p65*) relative expression exhibited an opposing trend (*p* < 0.05; Figure 8).

### 3.4. Muscle Color and Texture Characteristics

There was a linear increase in muscle yellowness (b*), springiness, and chewiness with increasing dietary MDA (*p* < 0.05). In contrast, muscle lightness (L*) and redness (a*) exhibited an inverse relationship (*p* < 0.05; Table 10). There was no significant difference in muscle hardness, resilience, cohesiveness, or gumminess of each group.

## 4. Discussion

MDA is a by-product of lipid peroxidation that has been considered a toxic and harmful substance [49]. Dietary inclusion of MDA-enriched oxidized fish oil has been documented to reduce the growth performance of hybrid grouper (*Epinephelus fuscoguttatus*♀ × *E. lanceolatus*♂) [12], juvenile Amur sturgeon (*Acipenser schrenckii*) [50], juvenile largemouth bass (*Micropterus salmoides*) [51], and tilapia (*Oreochromis niloticus*) [52]. The present study found that dietary MDA significantly increased feed conversion ratio (FCR) and decreased growth performance (FBW, WGR, SGR, PER, ILI, and ISI). The same results were observed in grass carp (*Ctenopharyngodon idellus*) [53] and hybrid grouper (*Epinephelus fuscoguttatus*♀ × *E. lanceolatus*♂) [54]. The reduction in growth performance observed in fish may be attributed to two primary factors: inhibited digestion and absorption of nutrients from the feed and a state of suboptimal health.

On the one hand, the growth of fish is contingent upon their ability to digest and absorb nutrients. Digestive enzymes break down nutrients into small molecules that can pass through the intestines and into the bloodstream, usually reflecting the ability of fish to digest nutrients [55]. The present study found that MDA markedly diminished the activities of pepsin, trypsin, lipase, and amylase. Yu et al. [52] found that MDA-enriched oxidized fish oil fed to farmed tilapia (*Oreochromis niloticus*) also exhibited inhibition of digestive enzyme activity. This may be due to the fact that MDA enters the fish and forms adducts with proteins and acetaldehyde in the intestine, thereby inhibiting the activity of protein-based digestive enzymes. Interestingly, different results were found in *Macrobrachium rosenbergii*, where protease activity was specifically increased. However, lipase activity had no significant effect [56]. Differences in feed composition (type and level of protein), oxidized fish oil composition (MDA levels), and farming cycles may account for this variation. Whole *Macrobrachium rosenbergii* samples were used for digestive enzyme analysis, which may also have contributed to the discrepancy. The proper development of the digestive tract is closely related to the digestion and absorption capacity of small molecules. Intestinal villi height reflects the contact area of fish with nutrients, while intestinal muscular layer thickness is related to the ability of intestinal peristalsis [57,58]. Indeed, HE staining of the stomach and intestines in this experiment was observed to coincide with reduced growth performance. The present study found that MDA significantly decreased stomach and intestinal villi height, villi width, and muscular layer thickness. The same results were found in hybrid groups [54]. This may be attributed to the fact that MDA possesses a markedly electrophilic 1,3-bis-unsaturated aldehyde structure, which effectively disrupts the cell membrane of the intestinal mucosa and facilitates its penetration into the cell. The intracellular presence of the compound may result in damage to the nucleus and disruption of the mitochondrial structure of the cell, thereby inhibiting the development and growth of the digestive tract [59,60].

On the other hand, the intestinal mucosal barrier plays a key role in the maintenance of fish health and normal growth. The integrity of the intestinal mucosal barrier can be assessed by measuring two key indicators: the DAO and LPS levels [61]. DAO is a cytoplasmic enzyme present in the epithelial cells of the intestinal villi and has a positive correlation with intestinal mucosal integrity [62]. LPS is mainly a component of the extracellular membrane of gram-negative bacteria and is normally found at low plasma levels [63]. The present study found that dietary MDA significantly increased serum DAO enzyme activity and LPS content. Similar results were found in grass carp (*Ctenopharyngodon idellus*) [53], which may be due to impaired intestinal barrier function and entry of substances from the intestinal lumen into the bloodstream. This hypothesis was further supported by the results of gastric SEM and intestinal TEM. The intestinal mucosal barrier serves as a primary defensive barrier of fish against external stimuli. Its protective function hinges on a complex network of biological, physical, chemical, and immune-related mechanisms [64,65]. Biological barriers are the first barrier system that MDA comes into contact with after it enters the organism. Intestinal microbial homeostasis is an inevitable condition for the health of the organism, and when the dynamic balance of the microbial is disrupted, it signifies that the organism is about to enter a non-healthy state [66]. In the present study, MDA altered the intestinal microbial structural composition. In addition, MDA disrupts the structure of dietary proteins, as reported in rice bran protein [17], rice protein [45], and walnut protein [67]. Thus, the main reason why MDA disrupts the balance of the intestinal microbial may be that it oxidizes feed proteins and alters the intestinal microbial living environment. Additionally, it has been reported that up-regulation of the relative expression of upper *occludin*, *claudin 12*, *claudin 7α*, and *ZO-2* refines fish epithelial tight junction structures [68]. The present study revealed that MDA down-regulated the relative expression of *occludin*, *claudin 12*, *ZO-2*, and *claudin 7α*, damaging the physical barrier of the intestine, which was consistent with the intestinal transmission electron microscopy observation. MDA regulates the glutathione/glutathione transferase pathway, and induction of apoptosis may lead to this result. The complement system is recognized as the nexus between innate and adaptive immunity [69]. The third and fourth components of complement (C3, C4) play key roles in the complement system [70]. Immunoglobulins are complex glycoproteins that recognize a variety of pathogens and recruit immune cells and factors to destroy them [71]. The present study found that feed MDA decreased intestinal C4 levels and IgM activity. Damage to the physical barrier of the intestine, increased permeability, harmful bacteria entering the blood system, and the greater probability of ectopic entry of MDA from the feed into the bloodstream may have contributed to this outcome. Inflammatory response and oxidative stress are two key factors in intestinal barrier damage that have been widely recognized [72,73]. Down-regulation of inflammatory factor (*il-8*, *p65*, *tnf-α*) relative expression and up-regulation of anti-inflammatory factor (*tgf-β1*, *il-10*) relative expression mitigate inflammatory responses in fish [74]. Antioxidant enzymes (POD, CAT, SOD, GPx, and GR) are the first line of defense against oxidative stress in fish [75]. The present study found that dietary MDA increased intestinal MDA levels, *il-8*, *p65*, and *tnf-α* relative expression levels, and was significantly negatively correlated with intestinal POD, CAT, SOD, and GR enzyme activities, and *tgf-β1*, *il-10* relative expression levels. This is a similar study in hybrid groups [54]. MDA enters the body to inhibit the Nrf2/Keap1 signaling pathway, suppresses related gene relative expression, and induces intestinal inflammatory response and oxidative stress, which are the possible reasons for this result. In summary, MDA damages the health of fish by decreasing the antioxidant capacity and inducing an intestinal inflammatory response, which in turn damages the intestinal mucosal barrier.

Muscle color is not only a measure of muscle health but also the most intuitive indicator of a fish’s price [76]. High-L* and low-b* in muscle color index are generally considered high-quality muscle [77]. The present study found a significantly higher b* in the dorsal muscle of the striped catfish. Zhang et al. [78] found that the dorsal and caudal b* of channel catfish (*Ictalurus punctatus*) were significantly increased after feeding MDA-enriched oxidized fish oil, which is consistent with the results of the present experiment. It is possible that MDA damages the intestinal mucosal barrier and then enters the muscle tissue along with the blood, reacts with proteins in the body in an adduct reaction, and releases free radicals to induce the oxidation of unsaturated fatty acids, which cannot be eliminated by autophagy of lysosomal enzymes, and is deposited as lipofuscin to appear as a yellow color [31,79]. In addition, Elevated b* has also been found to be associated with the accumulation of purple-red deoxymyoglobin and bright cherry-red Oxymyoglobin oxidized to tan Methemoglobin [80]. L* is positively correlated with the amount of α-actinin [81]. The present study found that MDA significantly reduced L*, an indicator of dorsal muscle coloration in striped catfish. However, L* of the color index was significantly increased when the yak sarcoplasmic protein was mixed with the MDA solution, contrary to the results of this experiment [82]. It is possible that the free water inside the myoplasmic protein emulsion causes a stronger light reflection effect, thus presenting a higher L* value as a reason for this phenomenon. Therefore, MDA increases the b* value of fish dorsal muscle and induces muscle yellowing, but the exact mechanism remains to be investigated. Constructing a bridge between MDA and the muscle yellowing problem is our forthcoming research.

## 5. Conclusions

In conclusion, high doses of MDA (5–80 mg/kg) reduced the growth performance of striped catfish. This was attributed to linear damage to the gastrointestinal tract, a linear decrease in antioxidant function, and the occurrence of an inflammatory response. High doses of MDA (>40 mg/kg) were observed to significantly increase dorsal muscle b-value and induce muscle yellowing. However, the mechanism remains to be further studied.

## Figures and Tables

**Table 1:** Ingredients and chemical compositions of the basal diet for striped catfish.

Ingredients	%	Proximate Composition	Content
White fish meal	15.00	Dry matter (DM, %)	89.53
Rapeseed meal	20.00	Crude protein (% DM)	33.80
Soybean meal	20.00	Crude lipid (% DM)	10.65
Wheat flour	15.00	Ash (% DM)	10.45
Rice bran	25.30	Gross energy (MJ/kg)	18.33
Soybean oil	2.00		
Ca(H_2_PO_4_)_2_	1.20		
Choline chloride (50%)	0.40		
Vitamin C	0.02		
Compound premix ^a^	1.00		
Cellulose microcrystalline	0.08		

^a^ Vitamin and mineral premixes included (g/kg mixture): vitamin A, 0.20 g; vitamin B_1_, 0.33 g; vitamin B_2_, 0.88 g; vitamin B_6_, 0.73 g; vitamin B_12_, 0.001 g; nicotinic acid, 2.89 g; vitamin D_3_, 0.003 g; vitamin K_3_, 0.66 g; vitamin E, 4.40 g; calcium pantothenate, 1.64 g; biotin, 0.003 g; folic acid, 0.07 g; vitamin C, 10.01 g; FeSO_4_·7H_2_O, 52.87 g; ZnSO_4_·7H_2_O, 43.15 g; MgSO_4_·H_2_O, 44.65 g; MnSO_4_·7H_2_O, 31.56 g; Ca(IO_3_)_2_, 0.42 g; H_3_ClCu_2_O_3_, 0.65 g; Na_2_SeO_3_, 0.11 g; CoCl_2_·6H_2_O, 0.14 g.

**Table 2:** Primers pair sequences used in real-time PCR.

Target Gene	Primer Sequence	bp	Accession No.
*ZO-2*	F: TGGGTGTGGTGAGCAGAGAGTC R: TGGTTGGTTGAAGTGTCCGTGTG	128	XM_034306344.1
*occludin*	F: ATGGAAGGTGTCATCGTGGTGTTG R: TGCCGAGACCGTACCCAGAAC	124	XM_034306959.1
*claudin 7α*	F: CGATGTTCTTGGCGGAGCTTTATTG R: CCTTGGTGCTGCTGGAAGTGC	101	XM_026935848.2
*Claudin 12*	F: CTCCAGCGGGTGTTACTACTTTGAC R: GCAGAGCCAAGCCAGAGAAGAAC	116	NM_001194860.2
*tnf-α*	F: TGTCTCGCTGGTCTGACTCCTATG R: CAGTGGGTTTGTTGCTCTTCAAGTG	97	XM_026942329.2
*p65*	F: GAATGCTGGGTGGAAGAGGAAGTG R: ATGGTCAGAGTGGCGGAAGAGG	115	XM_034301361.1
*il-8*	F: GCTTAGGGAGGTGAGGGCTGAG R: TAGGTGTGGAGGTGGATGTGGTAAG	112	XM_027138229.2
*il-10*	F: TCTACTTGGAGACCGTGTTGCCTAG R: GATGGTGTCGATGGGAGTTCTGAAG	80	XM_026935649.2
*tgf-β1*	F: GCTACTGTGGCACCTTATCAACCTG R: GCACCTCTTCTCCCTTTCCTTCATC	150	XM_026938647.2

*ZO-2*, zonula occludent 2; *tnf-α*, tumor necrosis factor alpha; *p65*, transcription factor p65; *il-8*, interleukin 8; *il-10*, interleukin 10; *tgf-β1*, transforming growth factor beta1; bp, base pairs.

**Table 3:** Growth parameters and body indexes of striped catfish-fed diets with various levels of malondialdehyde.

	Diets								Linear Regression Analysis		
	M0	M5	M10	M20	M40	M80	Pooled SEM	*p*-Value	Regression Equation	*p*-Value	R^2^
SGR (%/day)	2.18 ^a^	2.02 ^b^	2.00 ^b^	2.02 ^b^	2.08 ^ab^	2.08 ^ab^	0.02	0.001		ns	0.003
IBW (g)	31.45	31.40	31.29	31.39	31.41	31.34	0.02	0.447		ns	0.026
FBW (g)	106.56 ^a^	98.63 ^b^	97.46 ^b^	97.19 ^b^	98.92 ^b^	98.79 ^b^	0.82	<0.001		ns	0.089
WGR (%)	238.74 ^a^	214.07 ^b^	211.53 ^b^	209.36 ^b^	214.50 ^b^	215.30 ^b^	2.52	<0.001		ns	0.084
FCR	1.27 ^b^	1.43 ^a^	1.44 ^a^	1.45 ^a^	1.38 ^ab^	1.38 ^ab^	0.02	0.002		ns	0.002
PER	8.85 ^a^	7.79 ^b^	7.96 ^b^	7.60 ^b^	7.79 ^b^	7.75 ^b^	0.11	<0.001		ns	0.172
HSI (%)	1.63 ^b^	1.70 ^ab^	1.70 ^a^	1.71 ^a^	1.77 ^a^	1.90 ^a^	0.02	0.002	*y* = 0.003*x* + 1.657	<0.001	0.432
VSI (%)	11.19	11.66	11.35	11.58	10.66	11.20	0.22	0.843		ns	0.006
ILI (%)	213.43 ^a^	182.78 ^ab^	171.91 ^b^	170.60 ^b^	164.66 ^b^	168.42 ^b^	4.11	0.003	*y* = −0.315*x* + 1.685	0.018	0.103
ISI (%)	2.24 ^a^	1.80 ^b^	1.73 ^b^	1.75 ^b^	1.79 ^b^	1.66 ^b^	0.04	<0.001	*y* = −0.004*x* + 1.930	0.008	0.277
CF (g/cm^3^)	1.54	1.52	1.51	1.50	1.49	1.47	0.01	0.603		ns	0.083

Values are presented as means of triplication (*n* = 3); means with different superscript letters in the same row represented a significant difference (*p* < 0.05). Linear regression equation (where *x* is the level of malondialdehyde in diet and *y* is the response); R^2^, and *p*-Value are also given; ns, no significant. IBW, initial body weight; FBW, final body weight. Weight gain ratio (WGR) = 100 × (final body weight − initial body weight)/initial body weight; specific growth rate (SGR) = 100 × ((ln final body weight) − (ln initial body weight))/days; feed conversion ratio (FCR) = total feed intake (dry matter)/(final biomass − initial biomass + biomass of dead fish); protein efficiency ratio (PER) = (final body weight − initial body weight)/total protein intake; hepatosomatic index (HSI) = 100 × (hepatopancreas weight/whole body weight); viscerosomatic index (VSI) = 100 × (viscera weight/whole body weight); intestinal weight index (ISI) = 100 × (intestinal weight/whole body weight); intestinal length index (ILI) = 100 × (intestinal length/body length); condition factor (CF) = 100 × body weight/(body length)^3^.

**Table 4:** Stomach histomorphology of striped catfish-fed diets with various levels of malondialdehyde.

	Diets								Linear Regression Analysis		
	M0	M5	M10	M20	M40	M80	Pooled SEM	*p*-Value	Regression Equation	*p*-Value	R^2^
*Stomach* (μm)											
Villi width	116.42 ^a^	105.09 ^ab^	102.02 ^b^	101.25 ^b^	75.28 ^c^	73.97 ^c^	2.22	<0.001	*y* = −0.515*x* + 108.985	<0.001	0.572
Villi height	187.94 ^a^	179.61 ^ab^	179.93 ^ab^	176.15 ^ab^	171.15 ^b^	170.59 ^b^	1.61	0.014	*y* = −0.179*x* + 182.178	0.002	0.144
Muscular layer thickness	1005.17 ^a^	970.95 ^a^	966.05 ^a^	959.98 ^a^	862.83 ^b^	857.74 ^b^	10.18	<0.001	*y* = −1.860*x* + 985.159	<0.001	0.211
*Intestine* (μm)											
Villi width	82.31 ^a^	60.26 ^b^	59.01 ^b^	57.74 ^b^	57.24 ^b^	56.04 ^b^	1.19	<0.001	*y* = −0.178*x* + 66.709	<0.001	0.118
Villi height	508.71 ^a^	477.90 ^ab^	477.55 ^ab^	474.11 ^ab^	454.05 ^b^	460.37 ^b^	4.95	0.028	*y* = −0.455*x* + 487.195	0.011	0.046
Muscular layer thickness	65.09 ^a^	59.91 ^ab^	54.32 ^bc^	53.87 ^bc^	50.76 ^c^	50.51 ^c^	0.84	<0.001	*y* = −0.147*x* + 59.528	<0.001	0.173

Values are presented as means of triplication (*n* = 9); means with different superscript letters in the same row represented a significant difference (*p* < 0.05). Linear regression equation (where *x* is the level of malondialdehyde in diet and *y* is the response), R^2^, and *p*-Value are also given.

**Table 5:** Digestive enzyme activities of striped catfish-fed diets with various levels of malondialdehyde.

	Diets								Linear Regression Analysis		
	M0	M5	M10	M20	M40	M80	Pooled SEM	*p*-Value	Regression Equation	*p*-Value	R^2^
Pepsin (U/mg protein)	27.15	26.18	26.82	24.91	23.89	23.40	0.70	0.576		ns	0.200
Trypsin (U/mg protein)	501.58 ^a^	486.36 ^a^	435.33 ^ab^	380.95 ^b^	359.47 ^b^	362.81 ^b^	15.21	<0.001	*y* = −1.650*x* + 463.713	0.001	0.522
Lipase (U/mg protein)	123.28 ^a^	118.60 ^a^	105.34 ^ab^	98.26 ^ab^	77.11 b	73.78 ^b^	5.16	0.001	*y* = −0.621*x* + 115.441	<0.001	0.643
Amylase (U/mg protein)	0.94 ^a^	0.89 ^ab^	0.89 ^ab^	0.61 ^abc^	0.56 ^bc^	0.67 ^c^	0.04	0.001	*y* = −0.004*x* + 0.856	0.008	0.278
Maltase (U/mg protein)	233.22 ^a^	199.10 ^ab^	151.03 ^bc^	142.56 ^c^	117.12 ^c^	120.74 ^c^	10.96	<0.001	*y* = −1.149*x* + 190.321	0.001	0.488

Values are presented as means of triplication (*n* = 3); means with different superscript letters in the same row represented a significant difference (*p* < 0.05). Linear regression equation (where *x* is the level of malondialdehyde in diet and *y* is the response), R^2^, and *p*-Value are also given.

**Table 6:** Intestinal mucosal permeability of striped catfish-fed diets with various levels of malondialdehyde.

	Diets								Linear Regression Analysis		
	M0	M5	M10	M20	M40	M80	Pooled SEM	*p*-Value	Regression Equation	*p*-Value	R^2^
DAO (mU/L)	112.47 ^b^	128.59 ^b^	129.14 ^ab^	135.58 ^ab^	158.47 ^a^	158.51 ^a^	5.21	0.032	*y* = 0.533*x* + 123.350	0.002	0.465
LPS (ng/mL)	2.85 ^b^	3.32 ^ab^	3.57 ^ab^	3.49 ^ab^	4.08 ^ab^	4.79 ^a^	0.19	0.018	*y* = 0.022*x* + 3.126	<0.001	0.599

Values are presented as means of triplication (*n* = 3); means with different superscript letters in the same row represented a significant difference (*p* < 0.05). DAO, diamine oxidase; LPS, lipopolysaccharide. Linear regression equation (where *x* is the level of malondialdehyde in diet and *y* is the response), R^2^, and *p*-Value are also given.

**Table 7:** The richness and diversity analysis of intestinal contents in striped catfish-fed diets with various levels of malondialdehyde.

	Diets								Linear Regression Analysis		
	M0	M5	M10	M20	M40	M80	Pooled SEM	*p*-Value	Regression Equation	*p*-Value	R^2^
Goods_coverage	1.00	1.00	1.00	1.00	1.00	1.00	0.00	0.197		ns	0.343
Sob	369.00	393.67	511.00	495.00	592.33	481.67	27.03	0.458		ns	0.314
Shannon	4.64	3.27	4.30	4.39	5.00	4.85	0.17	0.259		ns	0.443
Chao1	409.05	439.41	557.73	542.61	649.03	534.80	28.24	0.568		ns	0.336
Simpson	0.92	0.72	0.82	0.89	0.91	0.93	0.02	0.070		ns	0.390
Ace	411.57	445.46	557.66	545.95	646.16	537.16	28.74	0.512		ns	0.324

Values are presented as means of triplication (*n* = 3); means with different superscript letters in the same row represented a significant difference (*p* < 0.05). Linear regression equation (where *x* is the level of malondialdehyde in diet and *y* is the response), R^2^, and *p*-Value are also given.

**Table 8:** Intestinal and plasma immunity factors of striped catfish-fed diets with various levels of malondialdehyde.

	Diets								Linear Regression Analysis		
	M0	M5	M10	M20	M40	M80	Pooled SEM	*p*-Value	Regression Equation	*p*-Value	R^2^
*Plasma*											
LYZ (U/L)	4.78	4.79	4.96	5.13	5.08	5.56	0.19	0.901		ns	0.102
C3 (μg/mL)	192.58	215.96	201.09	174.59	181.96	169.03	6.29	0.265		ns	0.207
C4 (μg/mL)	90.79	114.57	99.82	102.91	87.65	87.67	4.65	0.557		ns	0.085
IgM (mg/L)	36.77	31.52	47.01	37.85	37.95	37.00	1.48	0.051		ns	0.001
*Intestine*											
LYZ (U/g protein)	2.20	2.24	1.65	1.46	1.46	1.41	0.11	0.124	*y* = −0.008*x* + 1.915	0.034	0.253
C3 (mg/g protein)	41.59	36.53	36.40	29.88	24.07	21.65	2.46	0.106	*y* = −0.237*x* + 37.814	0.004	0.411
C4 (mg/g protein)	80.49 ^a^	77.06 ^a^	64.26 ^ab^	64.78 ^ab^	35.06 ^b^	42.02 ^b^	4.62	0.001	*y* = −0.510*x* + 73.794	0.001	0.541
IgM (mg/g protein)	17.02 ^a^	14.25 ^ab^	16.17 ^ab^	13.70 ^abc^	7.40 ^bc^	10.62 ^c^	0.92	0.002	*y* = −0.087*x* + 15.436	0.005	0.394

Values are presented as means of triplication (*n* = 3); means with different superscript letters in the same row represented a significant difference (*p* < 0.05). LYZ, lysozyme; C3, complement 3; C4, complement 4; IgM, immunoglobulin M. Linear regression equation (where *x* is the level of malondialdehyde in diet and *y* is the response), R^2^, and *p*-Value are also given.

**Table 9:** Intestinal antioxidant-related parameters of striped catfish-fed diets with various levels of malondialdehyde.

	Diets								Linear Regression Analysis		
	M0	M5	M10	M20	M40	M80	Pooled SEM	*p*-Value	Regression Equation	*p*-Value	R^2^
T-AOC (U/mg protein)	1.07	0.92	0.86	0.88	0.83	0.82	0.03	0.317		ns	0.166
POD (U/g protein)	54.22 ^a^	48.24 ^ab^	42.01 ^ab^	45.12 ^ab^	41.14 ^ab^	36.85 ^b^	1.73	0.030	*y* = −0.167*x* + 48.915	0.004	0.412
CAT (U/g protein)	16.96 ^a^	12.86 ^ab^	13.24 ^ab^	14.05 ^ab^	14.47 ^ab^	11.14 ^b^	0.56	0.038	*y* = −0.042*x* + 14.867	0.036	0.247
SOD (U/g protein)	63.56 ^a^	56.73 ^a^	55.25 ^a^	54.95 ^a^	29.85 ^b^	27.62 ^b^	3.80	0.001	*y* = −0.465*x* + 59.996	<0.001	0.662
GPx (U/g protein)	29.17 ^a^	23.30 ^b^	21.73 ^b^	21.61 ^b^	21.31 ^b^	21.96 ^b^	0.76	0.002		ns	0.176
GR (U/g protein)	24.79 ^a^	20.82 ^ab^	18.71 ^ab^	15.86 ^b^	15.16 ^b^	14.97 ^b^	0.99	0.007	*y* = −0.097*x* + 20.879	0.005	0.312
MDA (nmol/mg protein)	14.03 ^b^	13.71 ^b^	14.96 ^ab^	15.38 ^ab^	15.00 ^ab^	16.45 ^a^	0.26	0.038	*y* = 0.029*x* + 14.184	0.001	0.538

Values are presented as means of triplication (*n* = 3); means with different superscript letters in the same row represented a significant difference (*p* < 0.05). T-AOC, total antioxidant capacity; POD, peroxidase; CAT, catalase; SOD, superoxide dismutase; GPx, glutathione peroxidase; GR, glutathione reductase. Linear regression equation (where *x* is the level of malondialdehyde in diet and *y* is the response), R^2^, and *p*-Value are also given.

**Table 10:** Muscle color and texture characteristics of striped catfish-fed diets with various levels of malondialdehyde.

	Diets								Linear Regression Analysis		
	M0	M5	M10	M20	M40	M80	Pooled SEM	*p*-Value	Regression Equation	*p*-Value	R^2^
L*	63.00 ^a^	57.88 ^b^	57.41 ^b^	57.96 ^b^	55.45 ^b^	56.37 ^b^	0.48	<0.001	*y* = −0.053*x* + 59.390	0.002	0.131
a*	−1.08	−1.44	−1.40	−2.26	−2.02	−2.21	0.16	0.187	*y* = −0.012*x* − 1.421	0.041	0.101
b*	6.30 ^c^	6.97 ^bc^	7.20 ^bc^	8.23 ^bc^	9.29 ^b^	14.19 ^a^	0.49	<0.001	*y* = 0.095*x* + 6.236	<0.001	0.800
Resilience	0.05	0.05	0.05	0.05	0.05	0.05	0.00	0.446		ns	0.387
Springiness (mm)	0.21	0.24	0.24	0.24	0.27	0.32	0.02	0.291	*y* = 0.001*x* + 0.219	0.009	0.358
Cohesiveness	0.12	0.12	0.11	0.11	0.11	0.10	<0.01	0.790		ns	0.056
Chewiness (mJ)	52.97	54.33	66.86	70.12	89.68	124.98	58.94	0.078	*y* = 0.901*x* + 53.219	0.001	0.520
Gumminess (g)	208.33	233.13	252.85	235.25	273.53	263.28	10.81	0.579		ns	0.057
Hardness (kg)	2.24	2.12	2.08	2.07	2.39	2.21	0.05	0.444		ns	0.027

Values are presented as means of triplication (*n* = 3); means with different superscript letters in the same row represented a significant difference (*p* < 0.05). Linear regression equation (where *x* is the level of malondialdehyde in diet and *y* is the response), R^2^, and *p*-Value are also given. L*, lightness; a*, redness; b*, yellowness.

## Data Availability

Data are contained within the article.
